# Exposure to fluoride aggravates the impairment in learning and memory and neuropathological lesions in mice carrying the APP/PS1 double-transgenic mutation

**DOI:** 10.1186/s13195-019-0490-3

**Published:** 2019-04-22

**Authors:** Kun Cao, Jie Xiang, Yang-Ting Dong, Yi Xu, Yi Li, Hui Song, Xiao-Xiao Zeng, Long-Yan Ran, Wei Hong, Zhi-Zhong Guan

**Affiliations:** 1 Department of Pathology at the Affiliated Hospital of Guizhou Medical University, Guiyang, 550004 Guizhou People’s Republic of China; 2 Key Laboratory of Endemic and Ethnic Diseases of the Ministry of Education of P. R. China (Guizhou Medical University), Guiyang, 550004 Guizhou People’s Republic of China; 3 Key Laboratory of Medical Molecular Biology, Guiyang, 550004 Guizhou People’s Republic of China

**Keywords:** Alzheimer’s disease, APP/PS1 double-transgenic mice, Fluorosis, Learning and memory, Neuropathology

## Abstract

**Background:**

Alzheimer’s disease (AD) is responsible for 60–70% of all cases of dementia. On the other hand, the tap water consumed by hundreds of millions of people has been fluoridated to prevent tooth decay. However, little is known about the influence of fluoride on the expression of APP and subsequent changes in learning and memory and neuropathological injury. Our aim here was to determine whether exposure to fluoride aggravates the neuropathological lesions in mice carrying the amyloid precursor protein (APP)/presenilin1 (PS1) double mutation.

**Methods:**

These transgenic or wide-type (WT) mice received 0.3 ml of a solution of fluoride (0.1 or 1 mg/ml, prepared with NaF) by intragastric administration once each day for 12 weeks. The learning and memory of these animals were assessed with the Morris water maze test. Senile plaques, ionized calcium binding adaptor molecule 1 (Iba-1), and complement component 3 (C3) expression were semi-quantified by immunohistochemical staining; the level of Aβ42 was detected by Aβ42 enzyme-linked immunosorbent assays (ELISAs); the levels of synaptic proteins and enzymes that cleave APP determined by Western blotting; and the malondialdehyde (MDA) content and activities of superoxide dismutase (SOD) and glutathione peroxidase (GSH-Px) measured by biochemical procedures.

**Results:**

The untreated APP mice exhibited a decline in learning and memory after 12 weeks of fluoride treatment, whereas treatment of these some animals with low or high levels of fluoride led to such declines after only 4 or 8 weeks, respectively. Exposure of APP mice to fluoride elevated the number of senile plaques and level of Aβ42, Iba-1, and BACE1, while reducing the level of ADAM10 in their brains. The lower levels of synaptic proteins and enhanced oxidative stress detected in the hippocampus of APP mice were aggravated to fluoride.

**Conclusions:**

These findings indicate that exposure to fluoride, even at lower concentration, can aggravate the deficit in learning and memory and neuropathological lesions of the mice that express the high level of APP.

## Introduction

Alzheimer’s disease (AD), a neurodegenerative disorder characterized by progressive memory loss and other cognitive impairments [1], is responsible for 60–70% of all cases of dementia [2]. Extracellular senile plaques containing β-amyloid peptide (Aβ), intra-neuronal neurofibrillary tangles, brain atrophy, and loss of neurons are the neuropathological hallmarks of this disease [3, 4]. Clear evidence indicates that accumulation of Aβ, a 4-kDa polypeptide formed by proteolytic cleavage of amyloid precursor protein (APP) by β- and γ-secretases, is a primary pathogenic event [5, 6], leading to synaptic and neuronal loss, oxidative damage, and multiple inflammatory responses [7–9].

The several factors proposed to be mediators of AD pathogenesis include oxidative damage, inflammation, and synaptic disruption [10]. Early-onset AD is associated with accumulation of Aβ, which is thought to induce progressive synaptic damage [11, 12]. Moreover, Aβ disrupts the mitochondrial electron transfer chain [13], thereby increasing production of reactive oxygen species (ROS) [14] and impairing ATP synthesis [15].

Fluoride can cross both the blood-brain barrier and the plasma membrane of cells [16], allowing this ion to accumulate in the brain [17], where it can damage neurons [18]. Fluoride injures the central nervous system (CNS) by several mechanisms [18–20], in particular by elevating the level of oxidative stress [21–24]. Earlier studies in our own laboratory have documented direct toxic effects on the brains of experimental animals exposed to high levels of fluoride, including enhanced oxidative stress, reduction in the levels of nicotinic and muscarinic acetylcholine receptors, and mitochondrial abnormalities, along with impaired learning and memory [21, 24–27]. Moreover, fluoride increases lipid peroxidation and decreases the activity of antioxidant enzymes in rats, causing neurotoxicity, even in the second and third generations following exposure [28].

The tap water consumed by hundreds of millions of people has been fluoridated to prevent tooth decay [29], and fluoride has been added to toothpaste as well [30]. This is concerning in light of observations that either exposure to high levels of fluoride [27] or elevated expression of APP by mutation of APP or PS1 [31] can result in the brain damage, with attenuated learning and memory in rodents. However, at present, little is known about the influence of fluoride (especially in low amounts) on the expression of APP and subsequent changes in learning and memory, senile plaques, and other forms of neuropathological injury, which might be of importance in connection with the pathogenesis of AD.

Accordingly, the aim of the current investigation was to evaluate the effects of low doses of fluoride on the brains of mice carrying the APP/PS1 mutation, focusing on senile plaques, the enzymes that cleave APP, synaptic proteins, oxidative stress, and learning and memory.

## Materials and methods

### Materials

Sodium fluoride (Sigma-Aldrich, USA); mouse monoclonal anti-Aβ antibody (6E10, SIG-39340, BioLegend Inc., USA); rabbit polyclonal anti-C3 (complement component 3), rabbit polyclonal anti-Iba-1 (ionized calcium binding adaptor molecule 1), rabbit polyclonal anti-SNAP-25 (synaptosomal-associated protein 25) and mouse anti-BACE1 (β-site amyloid precursor protein cleavage enzyme 1) antibodies (GTX101316, GTX100042, GTX113839, and GTX78908, Gentex Inc., USA); mouse monoclonal anti-SYP (synaptophysin) and rabbit polyclonal anti-BACE2 antibodies (ab8049 and ab5670, Abcam Inc., USA); mouse monoclonal anti-ADAM10 (a disintegrin and metalloproteinase domain-containing protein 10) and mouse monoclonal anti-β-actin antibodies (sc-28358 and sc-376421, Santa Cruz Inc., USA); horseradish peroxidase-conjugated secondary antibody (7076s and 7074s, Cell signaling Inc., USA); kits for measuring malondialdehyde (MDA), superoxide dismutase (SOD) and glutathione peroxidase (GSH-Px) (A003-1, A001-1, and A005, Nanjing Jiancheng Bioengineering Institute, China); kits for measuring Aβ42 levels (KMB3441, Thermo Fisher Scientific, USA); and all other general chemicals (Sigma-Aldrich, USA) were purchased from the sources indicated.

### Experimental animals

**Fig. 1:**
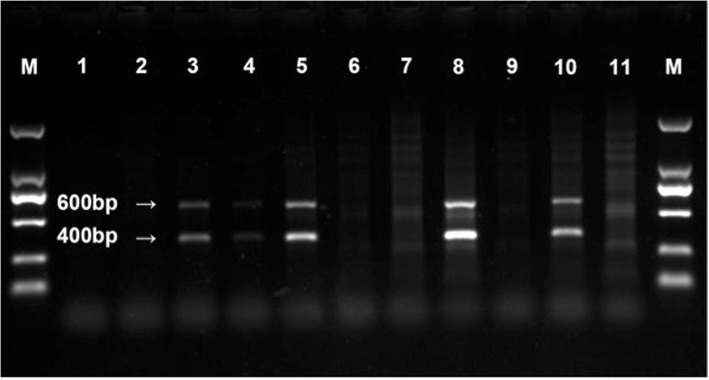
Confirmation of the genotype of the APP/PS1 double-transgenic mice. Bands 3, 5, 8, 10: the transgenic mice; bands: 1, 2, 4, 6, 7, 9, 11: wild-type mice. M, BioDL100 DNA markers

The APP/PS1 double-transgenic mice, B6.Cg-Tg (APPswe, PSEN1dE9) with a 85Dbo/Mmjax background, and the wild-type (WT) mice of this same strain used as controls were obtained from the Shanghai Research Center For Model Organisms, China. These animals were housed at 22–25 °C with a 12-h light/dark cycle and given free access to food and water in the SPF (specific pathogen-free) Animal Laboratory Center of Guizhou Medical University. The tip of the tail of each pup was cut off and DNA extracted for genotyping by the polymerase chain reaction (PCR), with detection of the products obtained by 1.5% agarose gel electrophoresis. The PCR primers for target transcripts (Table 1) were designed on the basis of the complete cDNA sequence deposited in GeneBank. When genomic DNA was utilized as the template for PCR amplification, products approximately 400 and 600 bp in size, consistent with the sizes of the APP and PS1 genes, respectively, were obtained with the transgenic, but not WT mice (Fig. 1).

**Table 1:** Sequences of the primers used to detect the APP and PS1 genes

Gene	Sequence	Length (bp)
APP	5′-gactgaccactcgaccaggttctg-3′	400
	5′-cttgtaagttggattctcatatccg-3′	
PS1	5′-aatagagaacggcaggagca-3′	608
	5′-gccatgagggcactaatcat-3′

Thirty double-transgenic mice at 3 months of age (half male and half female) and 30 age- and gender-matched WT mice were divided randomly into six groups (*n* = 10/per group) as follows: group I, untreated WT mice; group II, WT mice exposed to a lower concentration of fluoride (LF); group III, WT mice exposed to a higher levels of fluoride (HF); group IV, APP/SP1 transgenic mice (APP); group V, APP+LF; and group VI, APP+HF. The drinking water containing F^−^ was first prepared by dissolving NaF (analytical grade) in sterile double-distilled water (ddH_2_O) to obtain concentrations of 1 mg F^−^/ml (LF) or 10 mg F^−^/ml (HF) as the standard stock solution and then the stock solutions were diluted 10 times before feeding to mice. The mice in groups II and V were administered with 0.3 ml of the diluted LF solution; those in groups III and VI 0.3 ml of the diluted HF solution; and group I and IV 0.3 ml ddH_2_O by intragastric administration once a day for 12 weeks. The animal chow contained < 5 mg F^−^/kg.

During the experimental period, these animals performed the Morris water maze test once every 4 weeks. After 12 weeks, they were anesthetized and perfused with phosphate-buffered saline (PBS) via the jugular vein, and tissue samples were removed and stored at − 80 °C until analysis. The experiments described here were pre-approved by the Ethical Committee of Guizhou Medical University, China (No. 1702110).

### Determination of fluoride levels in the bone and brain

In the case of bone, adhering soft tissue was removed with a razor blade, the tissue then pulverized into a fine powder, and 1 mg analyzed. The brain tissue was homogenized in ice-cold PBS, the resulting homogenate centrifuged at 3000 rpm for 15 min, and the supernatant thus obtained used for analysis. In both cases, the level of fluoride was determined with a fluorine ion-selective electrode.

### The Morris water maze test of spatial learning and memory

Each mouse was forced to find a submerged escape platform in a circular pool filled with water (25–26 °C) rendered opaque (white) with powdered milk. During the familiarization session and acquisition phase (four trials/day for four consecutive days), the mouse was given as long as 60 s to find the hidden platform and then required to remain seated on this platform for 5 s, after which it was returned to its home cage. During the retention phase, the platform was removed from the pool and for 60 s the path taken by each mouse was video-filmed to determine the time required to swim to the original position of the platform as well as the number of passes over and time spent at this position.

### Immunohistochemical staining

Immunohistochemical staining for Aβ in the brain was performed as described previously [32]. Sections were first dehydrated in xylene, then rehydrated through a graded series of alcohol and rinsed with PBS; next, microwaved in 0.01 M citric acid buffer (pH 6.0) for 20 min to achieve antigen retrieval; and, following three washes with PBS, placed in blocking buffer (DAKO) for 30 min at room temperature (RT). Subsequently, the sections were incubated with 6E10 (diluted 1:100), anti-Iba-1 (diluted 1:500), and anti-C3 antibodies (diluted 1:300) at 4 °C overnight, and the next day, with secondary antibody, (i.e., biotinylated goat anti-mouse IgG) (diluted 1:200 in PBST) for 60 min at RT. Subsequently, they were incubated with the avidin-biotinylated enzyme complex and then placed in peroxidase reaction solution containing diaminobenzidine (DAB). As a negative control, the sections were processed in the same manner except without the primary antibody.

The senile plaques and the positive area % of immunohistochemical staining for Iba-1/C3 in randomly selected fields of vision (original magnification × 100 or × 200) were counted with the Image Pro Plus software (Image Pro Plus software, USA).

### Measurement of Aβ42 level

Tissues of the cortex were collected as described above. Concentration of Aβ42 in mouse brains was detected using an Amyloid β42 Mouse ELISA kit (KMB3441, Thermo Fisher Scientific, Inc.) according to the manufacturer’s protocol. Aβ42 content was examined by extraction of homogenate pellets in 5 M guanidine-HCl. Briefly, equal amounts of protein in each group were diluted to 100 μl volume and incubated in plate wells at room temperature for 2 h. The plate wells were then washed and incubated with 100 μl of Detection Antibody solution for 1 h at room temperature. Afterwards, the wells were washed and incubated with 100 μl of HRP-linked antibody solution for 30 min followed by four times of wash. Finally, the optical density was read at 450 nm on a spectrophotometer (Bio-Rad) after the adding of Stop Solution.

### Quantification of enzymes that cleave APP and synaptic proteins by Western blotting

The levels of enzymes that cleave APP (BACE1, a β-secretase; ADAM10, a constitutive α-secretase; and BACE2, another β-secretase) and of synaptic proteins (SNAP25 and SYP) were determined by Western blotting. For this purpose, the brain tissues were homogenized in PBS containing complete protease inhibitors in a glass vesicle; the resulting homogenate was centrifuged at 12,000 rpm at 4 °C for 20 min; and the protein concentrations of the supernatant thus obtained determined with the BCA protein assay kit, and the protein sample of each group was 30 μg.

The proteins were subsequently separated by 10% SDS-PAGE and then blotted onto polyvinylidene difluoride (PVDF) membranes with a transfer unit (Bio-Rad Inc.). For the relative quantification of the proteins, these membranes were thereafter incubated with antibody against BACE1 (1:1000 dilution), ADAM10 (1:1500 dilution), BACE2 (1:500, dilution), SNAP-25 (1:1000 dilution), SYP (1:500 dilution), or β-actin antibody (1:5000, dilution), at 4 °C overnight. After washing, the membranes were incubated with horseradish peroxidase-conjugated secondary antibody (1:5000) for 60 min. Finally, these membranes were incubated in ECL Plus reagent, and the signals thus obtained visualized by exposure to hyper-performance chemiluminescence film for 30 s to 5 min.

### Biochemical assay of the content of MDA and activities of SOD and GSH-PX

After homogenizing the brain tissues in ice-cold PBS and centrifuging homogenate at 5000 rpm for 10 min at 4 °C, the content of MDA and activities of SOD and GSH-PX in the supernatant were determined by appropriate kits, in accordance with the manufacturers’ instructions.

### Statistical analyses

The values for the different groups of mice are expressed as means ± SD and compared by analysis of variance (ANOVA), followed by a least significant differences post hoc test. Semi-quantitation of the Aβ plaques was carried out with the Bielschowsky procedure [33]. Differences with a value *P* < 0.05 were considered to be statistically significant. All analyses were performed using the SPSS 22.0 software (SPSS Inc., USA).

## Results

### The primary toxic manifestations of fluorosis

**Fig. 2:**
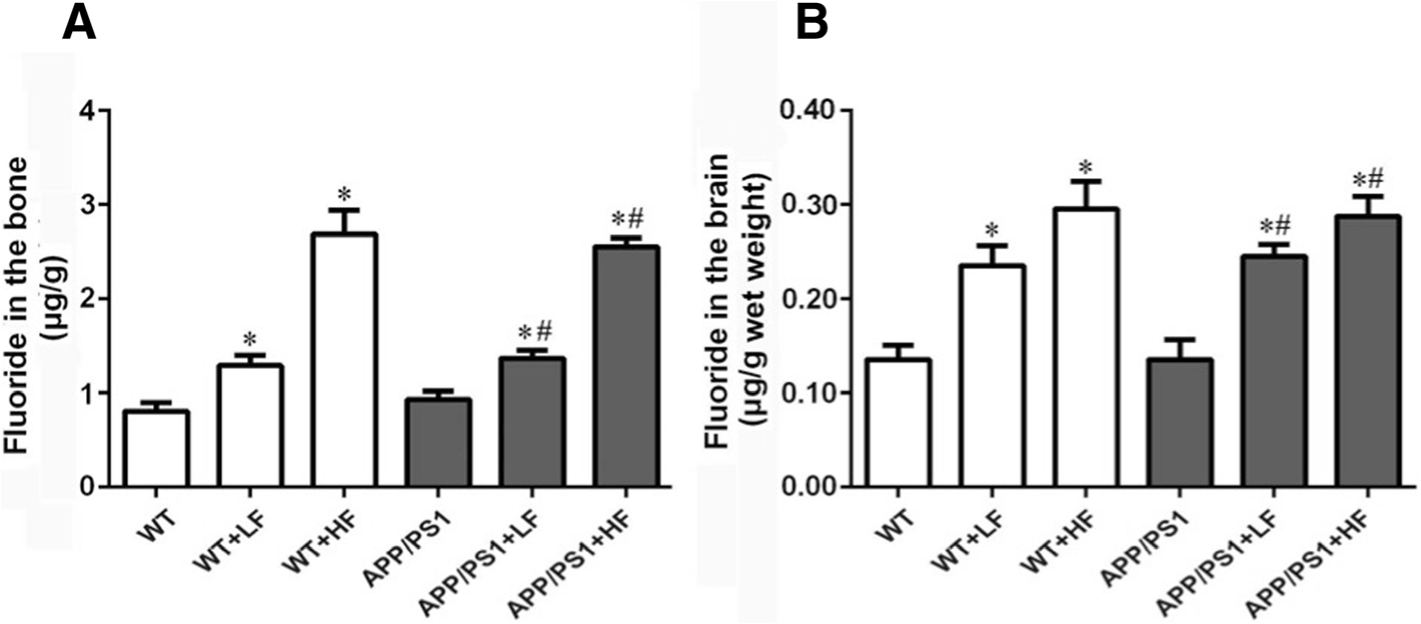
**a**, **b** Fluoride contents of the bone and brain of the mice in the different groups after 12 weeks of treatment. WT, wild-type; LF, low fluoride; HF, high fluoride. The values presented are means ± SD (*n* = 10). *P < 0.05 in comparison to WT; ^#^*P* < 0.05 in comparison to the APP/PS1 mice

Dental fluorosis is a sign of fluoride poisoning which appear as white or pigmented bands on the teeth (I°) and/or gray enamel (II°) or even loss of tooth structure (III°). Ten to twenty percent of our mice exhibited slight of dental fluorosis (I°), while all of the HF mice demonstrated obvious dental fluorosis (Table 2). Moreover, the fluoride contents of the bone and brain were increased significantly in all animals exposed to fluoride (Fig. 2a, b)

**Table 2:** Dental fluorosis in the different groups of mice

Group	Numbers	Dental fluorosis		
		I^°^	II^°^	III^°^
WT	10	0	0	0
WT+LF	10	1	0	0
WT+HF	10	5	5	0
APP/PS1	10	0	0	0
APP/PS1+LF	10	2	0	0
APP/PS1+HF	10	6	4	0

Notice: *WT*, wild-type; *LF*, low fluoride; *HF*, high fluoride; *APP/PS1*, amyloid precursor protein/presenilin 1, double mutation. I°: showing in form of white or pigmented bands; II°: gray enamel; III°: loss of tooth structure

### Spatial learning and memory of mice

**Fig. 3:**
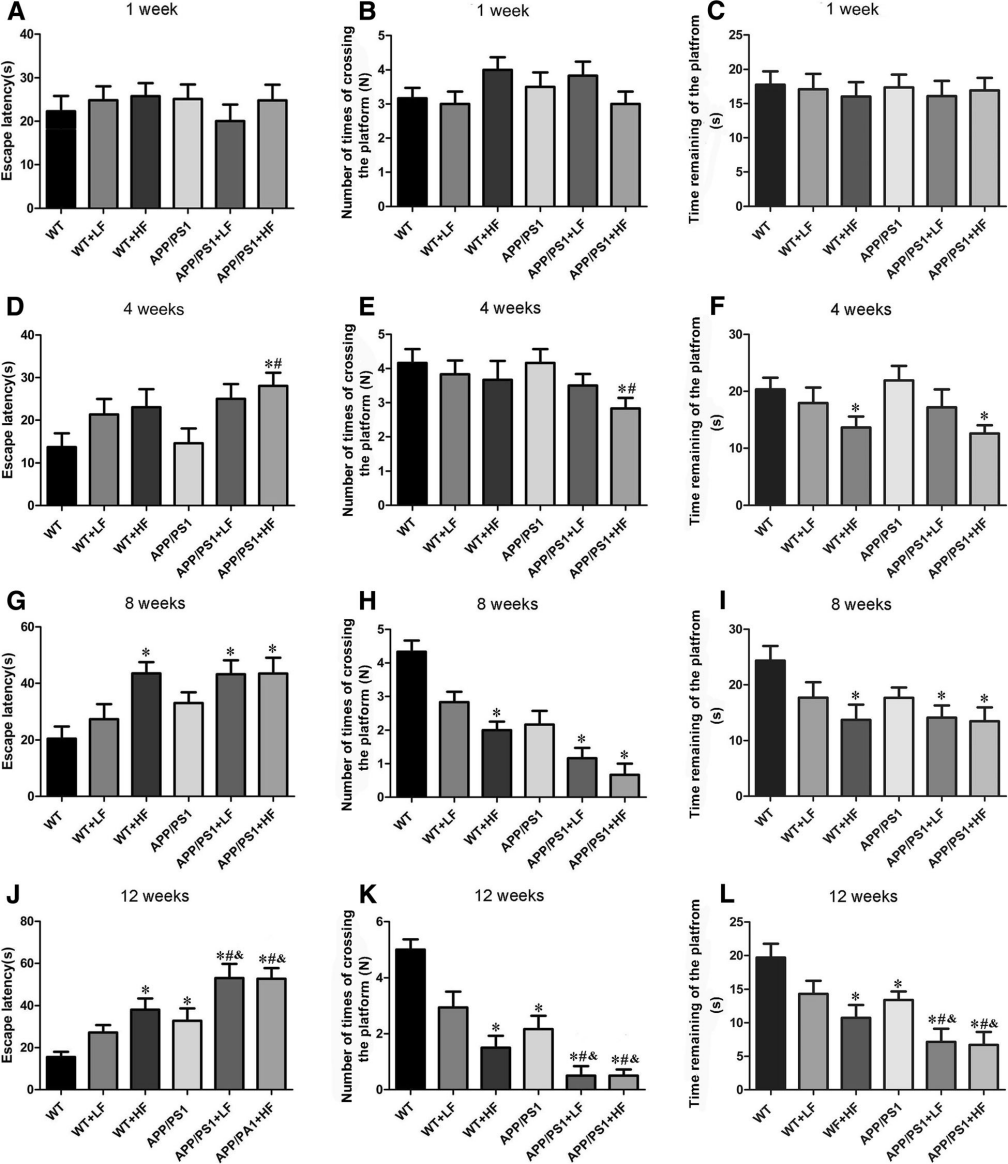
Effects of fluoride on the learning and memory of mice as assessed by the Morris water maze test. Exposure for 1 (**a**–**c**), 4 (**d**–**f**), 8 (**g**–**i**) and 12 weeks (**j**–**l**), respectively. WT, wild-type; LF, low fluoride; HF, high fluoride. The values shown are means ± SD (*n* = 10). *P < 0.05 in comparison to WT; ^#^*P* < 0.05 in comparison to APP/PS1 mice; ^&^*P* < 0.05 in comparison to WT+LH

After 1 week of exposure to fluoride, there were no significant differences in the escape latency, number of platform crossings, and time spent at the original position of the platforms between the different groups of mice (Fig. 3a, b). After 4 weeks, the learning and memory of the mice in group VI (APP+HF) and the time at the platform for group III (WT+HF) were reduced (Fig. 3d–f). After 8 weeks, learning and memory in the HF and APP+LF mice were impaired as compared the corresponding controls (Fig. 3g–i). After 12 weeks, learning and memory was impaired in the HF and APP mice and even more so in the APP animals exposed to high or lower levels fluoride (Fig. 3j–l).

### Numbers of senile plaques in the brain

**Fig. 4:**
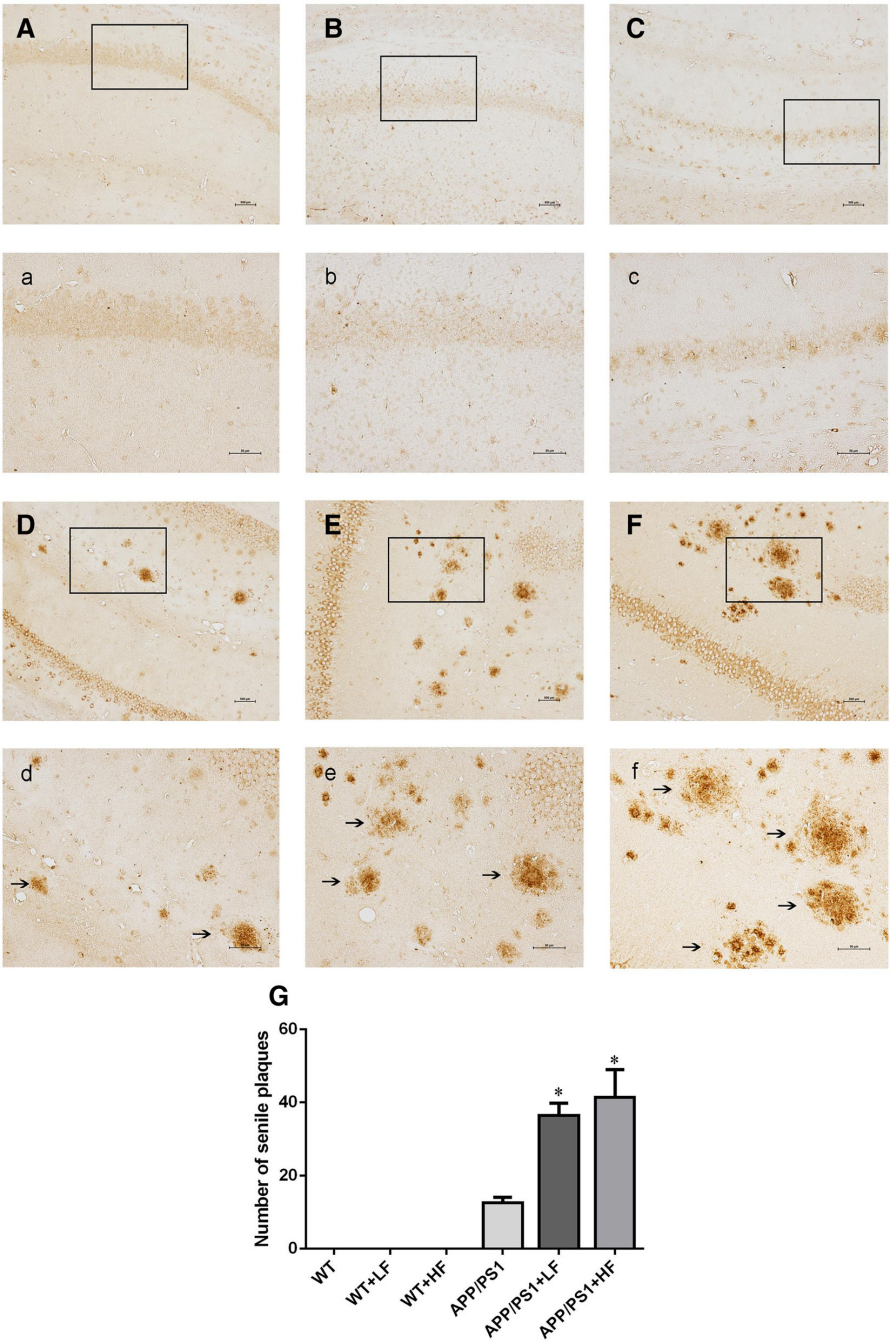
Senile plaques in the hippocampus of APP/PS1 mice following 12 weeks of exposure to fluoride. WT, wild-type; LF, low fluoride; HF, high fluoride. **A**, **a** WT mice without exposure. **B**, **b** WT receiving LF. **C**, **c** WT receiving HF. **D**, **d** APP/PS1 mice without exposure. **E**, **e** APP/PS1 mice receiving LF. **F**, **f** APP/PS1 receiving HF. Magnification: **A**–**F**, × 100, scale bar = 500 μm; **a**–**f**, × 200, scale bar = 50 μm. The solid arrows indicate senile plaques. **G** The numbers of senile plaques detected by 6E10 immunohistochemistry. The values shown are the means ± SD (*n* = 10). *P < 0.05 compared to APP/PS1 mice, as determined by analysis of variance (ANOVA), followed by the least significant differences post hoc test

When we explored whether fluoride can influence the neuropathological changes observed in mice expressing APPat high levels, immunohistochemical staining of the senile plaques in the hippocampus of these APP/PS1 mice (Fig. 4D–F) revealed significantly higher numbers of senile plaques in animals exposed to high or lower levels of fluoride (Fig. 4E, e, F, f). Similar differences were also seen in the cortex (not shown).

### Levels of Iba-1 and C3 proteins in the brain

**Fig. 5:**
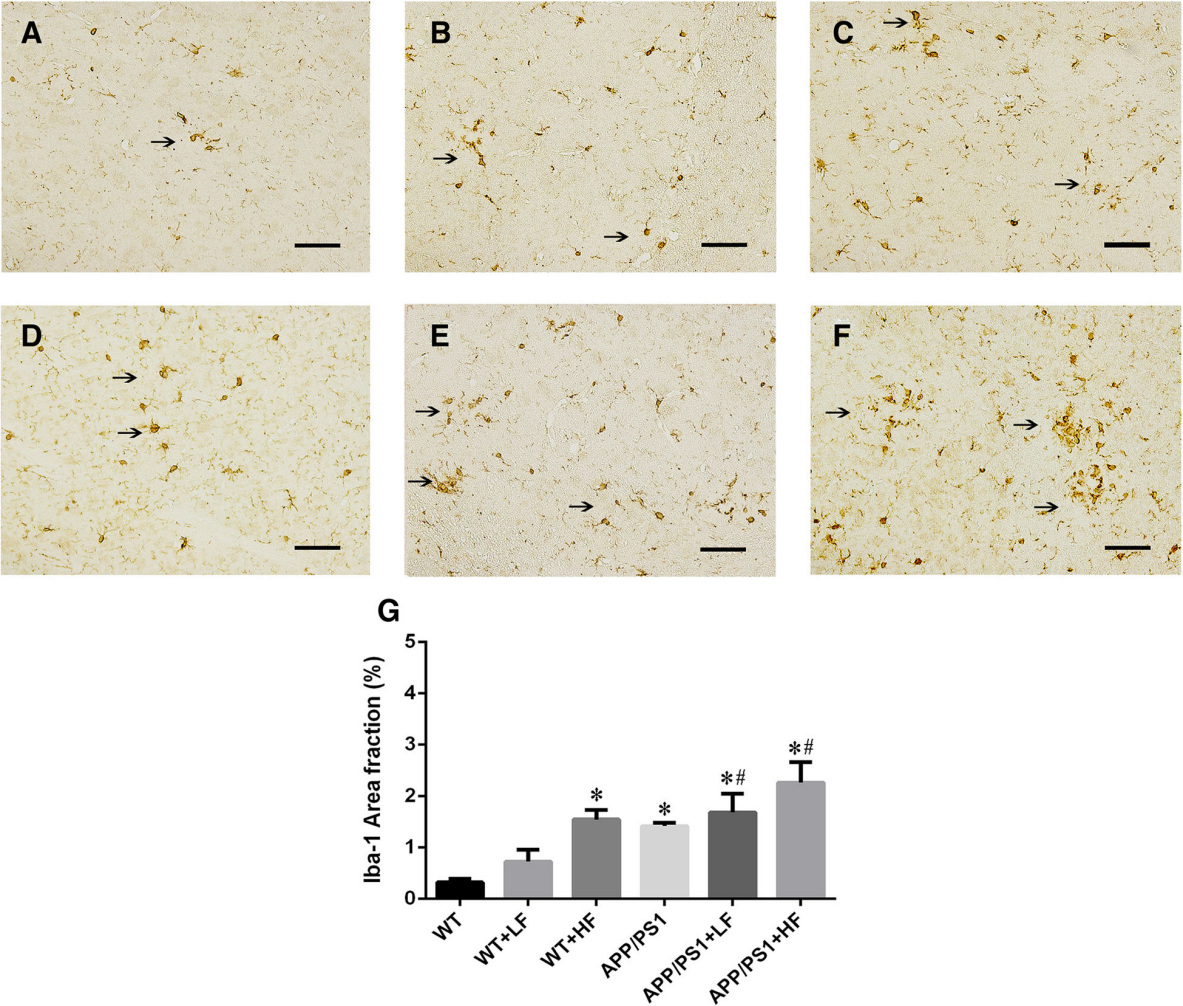
Immunohistochemical staining for Iba-1 in the brains of wild-type and APP/PS1 mice after 12 weeks of exposure to fluoride. WT, wild-type; LF, low fluoride; HF, high fluoride. **a** WT mice without exposure. **b** WT receiving LF. **c** WT receiving HF. **d** APP/PS1 mice without exposure. **e** APP/PS1 mice receiving LF. **f** APP/PS1 receiving HF. Magnification: **a**–**f**, × 200, scale bar = 50 μm. **g** Positive area % of Iba-1 immunostaining detected by immunohistochemistry. The values shown are the means ± SD (*n* = 10). *P < 0.05 compared to APP/PS1 mice; ^#^*P* < 0.05 in comparison to APP/PS1 mice, as determined by analysis of variance (ANOVA), followed by the least significant differences post hoc test

**Fig. 6:**
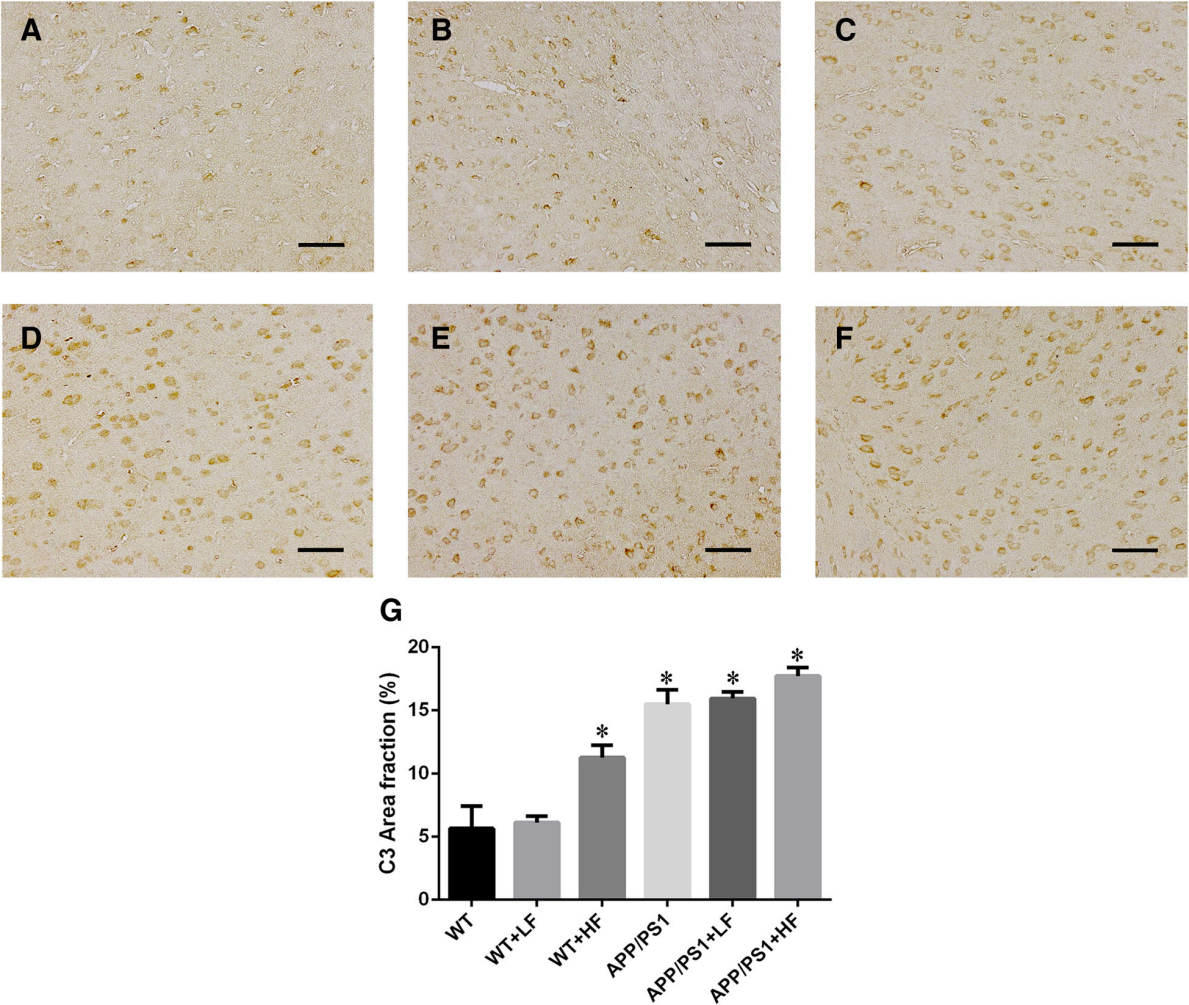
Immunohistochemical staining for C3 in the brains of wild-type and APP/PS1 mice after 12 weeks of exposure to fluoride. WT, wild-type; LF, low fluoride; HF, high fluoride. **a** WT mice without exposure. **b** WT receiving LF. **c** WT receiving HF. **d** APP/PS1 mice without exposure. **e** APP/PS1 mice receiving LF. **f** APP/PS1 receiving HF. Magnification: **a**–**f**, × 200, scale bar = 50 μm. **g** Positive area % of Iba-1 immunostaining detected by immunohistochemistry. The values shown are the means ± SD (*n* = 10). *P < 0.05 compared to APP/PS1 mice, as determined by analysis of variance (ANOVA), followed by the least significant differences post hoc test

The analysis of positive immunostaining for Iba-1 mainly detected in the cytoplasm (Fig. 5a–f) showed that the increased positive area % of Iba-1 immunostaining was observed in WT mice exposed to high fluoride and the mice carrying the APP/PS1 mutation. In addition, this raise of Iba-1 protein was augmented in the APP/PS1 mutation mice exposed to lower or high fluoride (Fig. 5g). However, the expression of complement C3 in the mice carrying the APP/PS1 mutation did not differ from the mice in other groups (Fig. 6a–g).

### Alterations in the concentration of Aβ42 in the mouse brain cortex

**Fig. 7:**
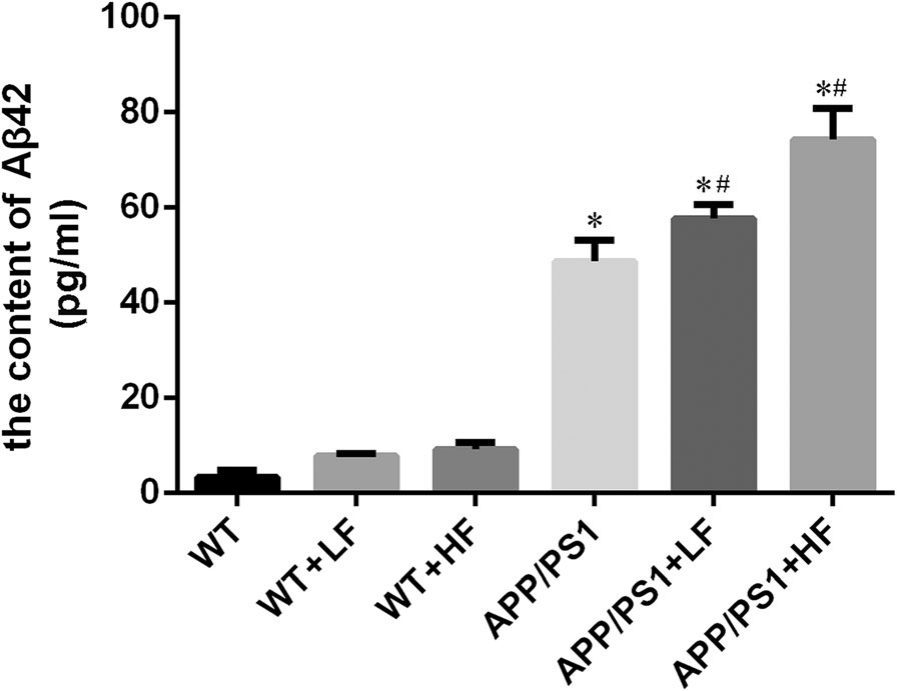
The content of Aβ42 in the brains of wild-type and APP/PS1 mice after 12 weeks of exposure to fluoride. WT, wild-type; LF, low fluoride; HF, high fluoride. The contents were determined by Aβ42 ELISA assay, and the values presented are means ± SD (*n* = 10). *P < 0.05 in comparison to WT control; ^#^*P* < 0.05 in comparison to APP/PS1 mice, as determined by analysis of variance (ANOVA), followed by the least significant differences post hoc test

The concentration of Aβ42 in the cortex of mouse brains did not differ significantly between the different groups of WT mice with or without the exposure of fluoride, but significantly increased in all of the APP/PS1 mice as comparison with WT. Furthermore, the concentration of Aβ42 in the mice with APP/PS1 mutation exposed to fluoride was significantly higher than those with APP/PS1 mutation without exposure of fluoride (Fig. 7).

### Levels of enzymes that cleave APP in the brain

**Fig. 8:**
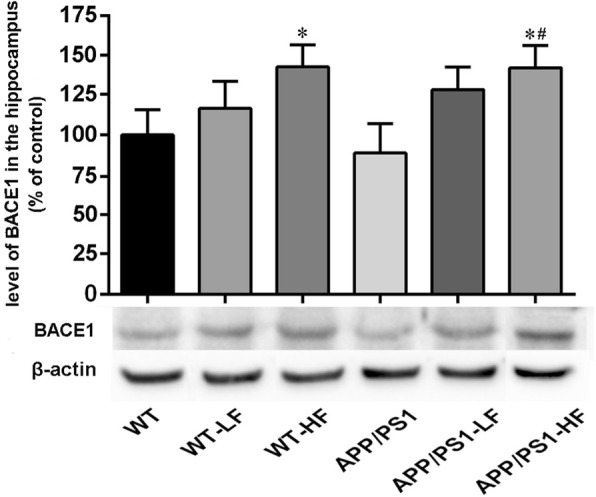
Expression of BACE1 in the hippocampus of the brains of wild-type and APP/PS1 mice after 12 weeks of exposure to fluoride. WT, wild-type; LF, low fluoride; HF, high fluoride. The levels were determined by Western blotting and the values presented are means ± SD (*n* = 10). *P < 0.05 in comparison to WT control; ^#^*P* < 0.05 in comparison to APP/PS1 mice, as determined by analysis of variance (ANOVA), followed by the least significant differences post hoc test

**Fig. 9:**
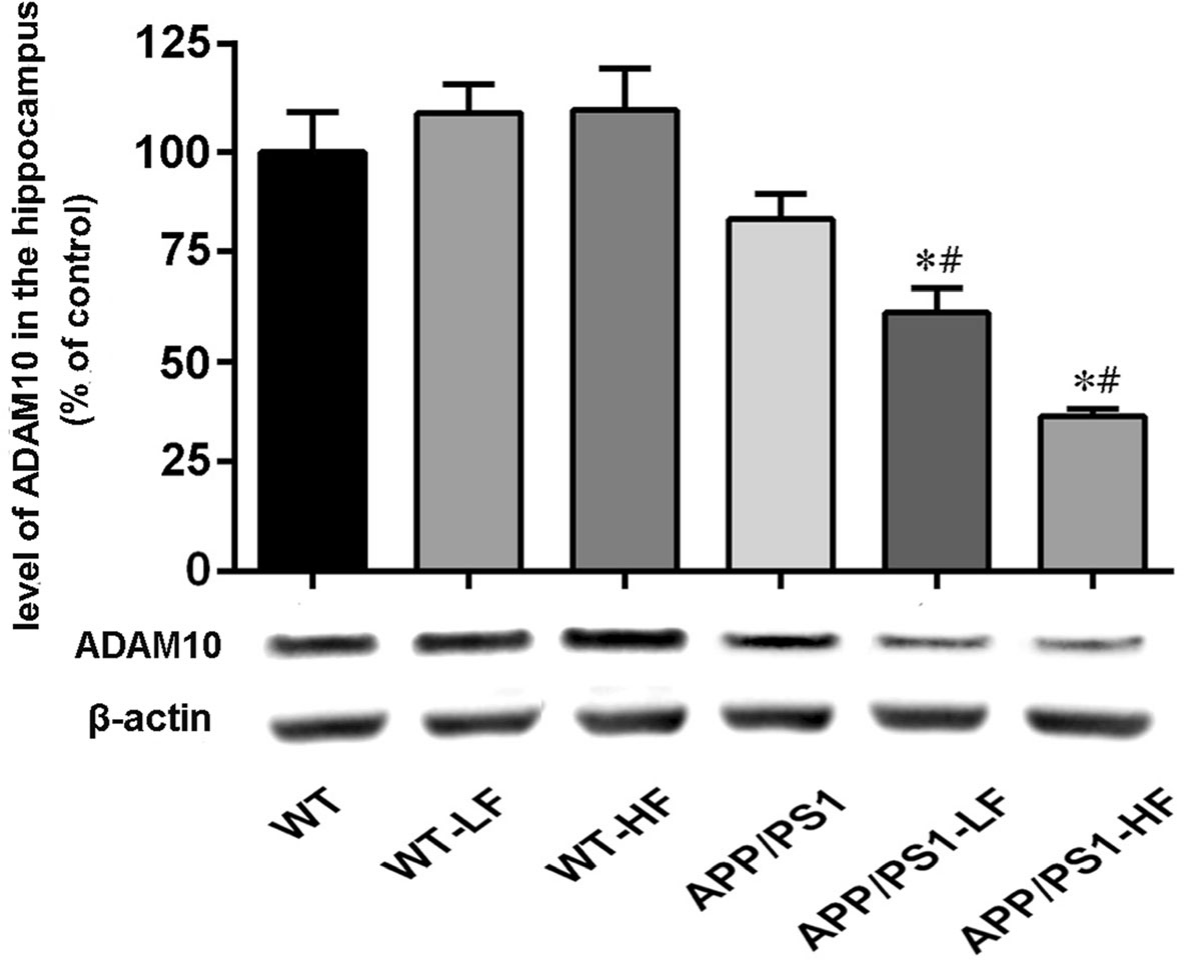
Expression of ADAM10 in the hippocampus of the brains of wild-type and APP/PS1 mice after 12 weeks of exposure to fluoride. WT, wild-type; LF, low fluoride; HF, high fluoride. The levels were determined by Western blotting and the values presented are means ± SD (*n* = 10). *P < 0.05 in comparison to WT control; ^#^*P*<0.05 in comparison to APP/PS1 mice, as determined by analysis of variance (ANOVA), followed by least significant differences post hoc test

**Fig. 10:**
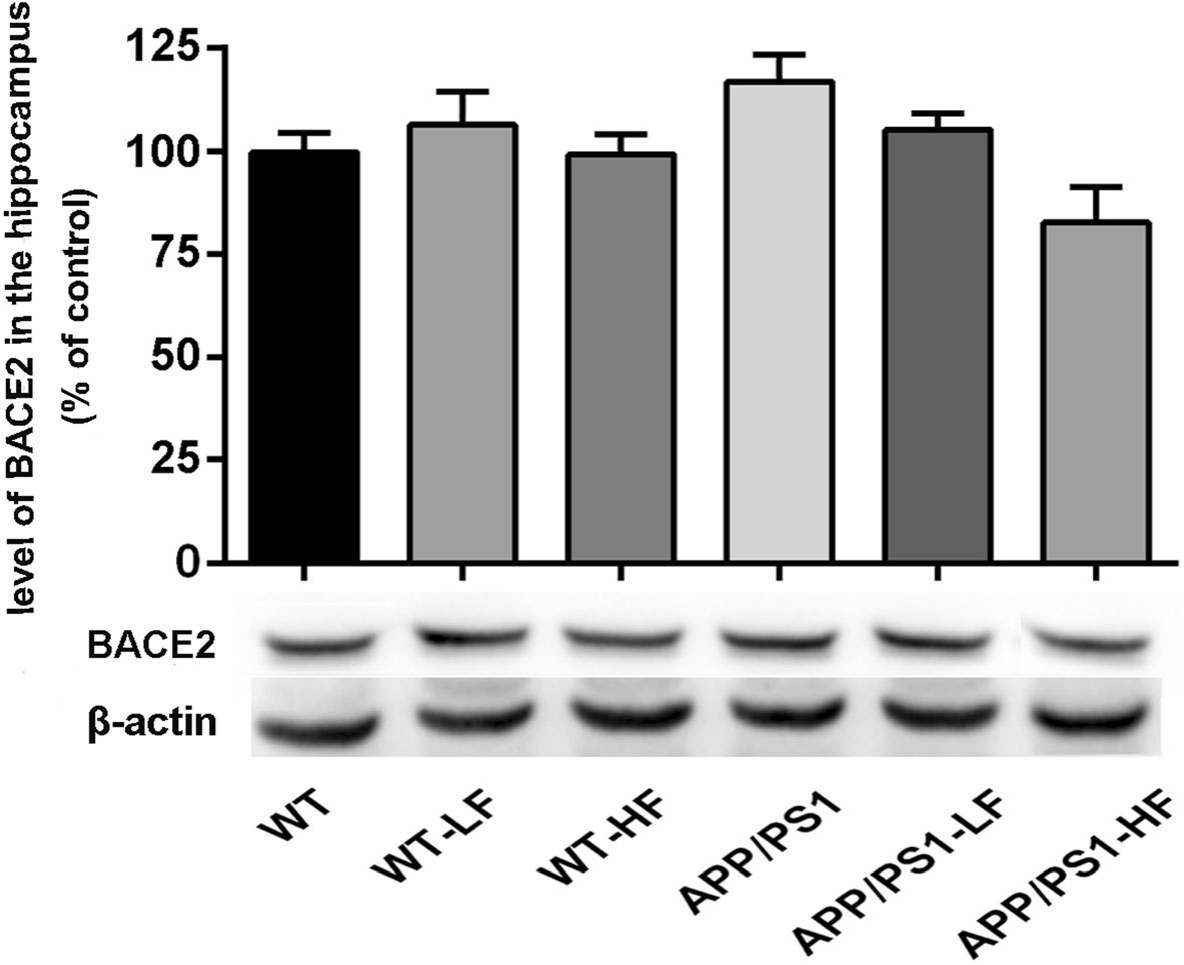
Expression of BACE2 in the hippocampus of the brains of wild-type and APP/PS1mice after 12 weeks of exposure to fluoride. WT, wild-type; LF, low fluoride; HF, high fluoride. The levels were determined by Western blotting and are the values presented are means ± SD (*n* = 10). *P < 0.05 in comparison to WT control; ^#^*P*<0.05 in comparison to APP/PS1 mice, as determined by analysis of variance (ANOVA), followed by least significant differences post hoc test

In comparison to untreated controls, the level of BACE1 was significantly higher in the brains of mice exposed to high fluoride or carrying the APP/PS1 mutation alone (Fig. 8), while the level of ADAM10 was reduced in these same animals (Fig. 9). The level of BACE2 in the brain did not differ significantly between the different groups of mice (Fig. 10).

### Levels of synaptic proteins in the brain

**Fig. 11:**
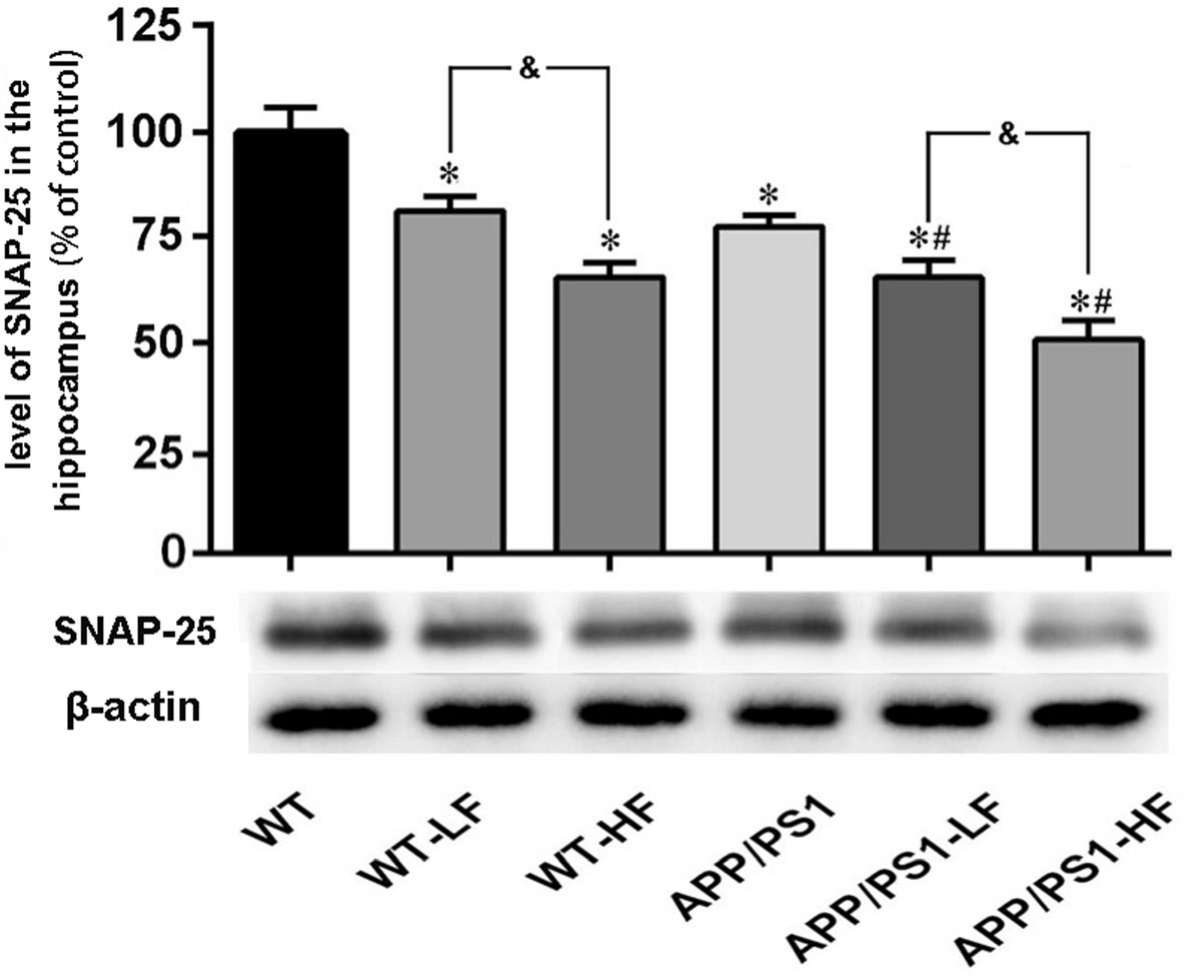
Expression of SNAP-25 in the hippocampus of the brains of wild-type and APP/PS1mice after 12 weeks of exposure to fluoride. WT, wild-type; LF, low fluoride; HF, high fluoride. The levels were determined by Western blotting and the values presented are means ± SD (*n* = 10). *P < 0.05 in comparison to WT control; ^#^*P*<0.05 in comparison to APP/PS1 mice; ^&^*P*<0.05 in comparison to WT+LF or APP/PS1+LF, as determined by analysis of variance (ANOVA), followed by the least significant differences post hoc test

**Fig. 12:**
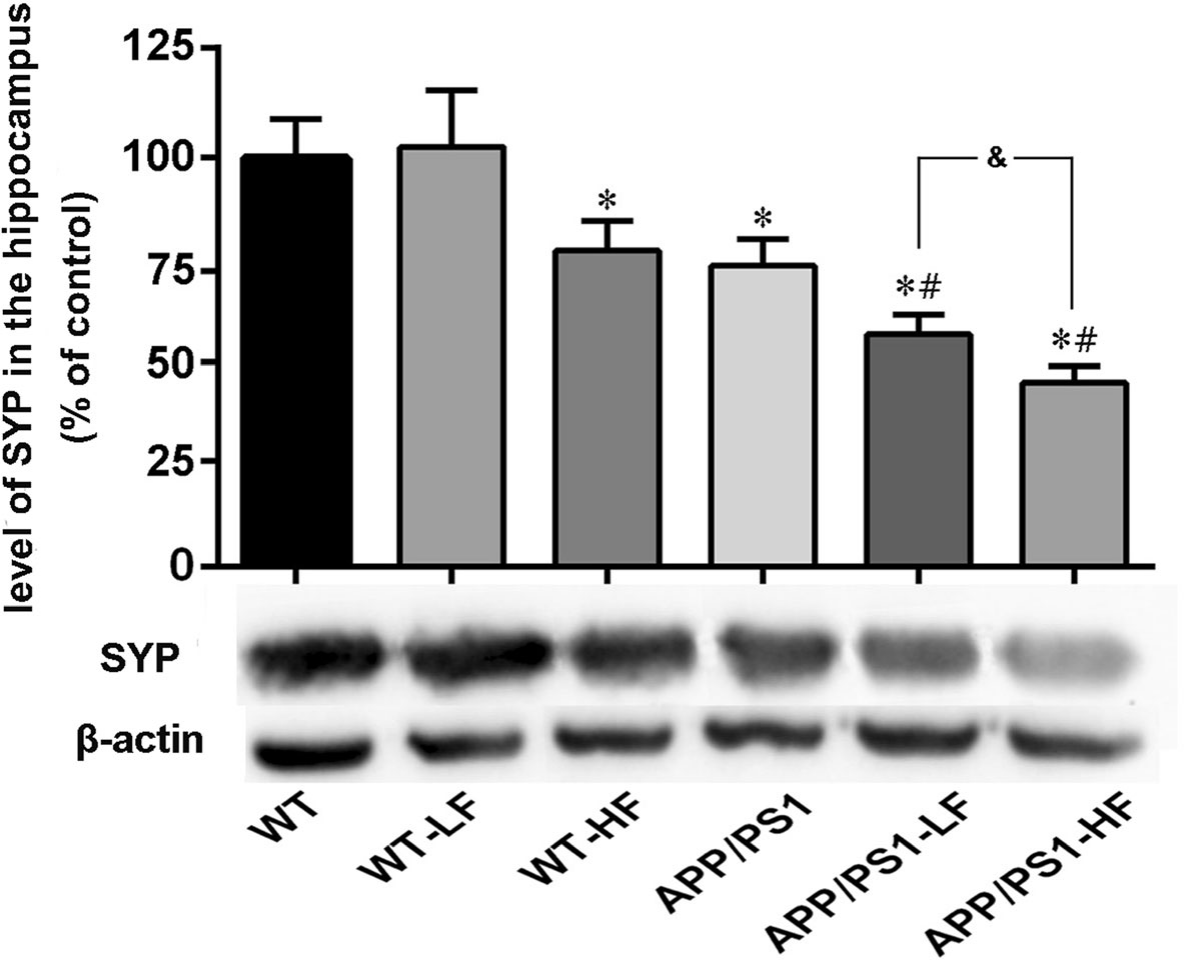
Expression of SYP in the hippocampus of the brains of wild-type and APP/PS1mice after 12 weeks of exposure to fluoride. WT, wild-type; LF, low fluoride; HF, high fluoride. The levels were determined by Western blotting and the values presented are means ± SD (*n* = 10). *P < 0.05 in comparison to WT control; ^#^*P* < 0.05 in comparison to APP/PS1 mice; ^&^*P*<0.05 in comparison to APP/PS1+LF, as determined by analysis of variance (ANOVA), followed by the least significant differences post hoc test

The levels of SNAP-25 (Fig. 11) and SYP (Fig. 12) proteins were significantly lower in the brains of WT mice exposed to either high or lower fluoride. These levels were also lower in the mice carrying the APP/PS1 mutation; furthermore, with exposure of fluoride to the animals this decline of synaptic proteins was augmented.

### The content of MDA and activities of SOD and GSH-Px in the brain

The level of MDA in the brains of mice with fluorosis or carrying the APP/PS1 mutation were significantly higher than those in the corresponding control groups, whereas the activities of SOD and GSH-Px were clearly lower (Table 3). Exposure to fluoride raised the level of oxidative stress in the brains of the APP/PS1 mice (Table 3).

**Table 3:** The MDA content and activities of SOD and GSH-Px in the brains of mice following 12 weeks of exposure to fluoride

Group	*n*	MDA (nmol/mg pr)	SOD (U/mg pr)	GSH-Px (kU/l)
WT	10	2.36 ± 0.12	206.85 ± 15.97	334.88 ± 5.53
WT+LF	10	2.60 ± 0.19	185.16 ± 16.01^*^	314.15 ± 12.5^*^
WT+HF	10	5.36 ± 0.29^*#^	101.32 ± 12.41^*#^	139.54 ± 11.28^*#^
APP/PS1	10	4.77 ± 0.13^*^	124.14 ± 7.71^*^	189.91 ± 5.43^*^
APP/PS1+LF	10	6.79 ± 0.43^*^^	81.55 ± 4.43^*^^	126.05 ± 8.87^*^^
APP/PS1+HF	10	7.47 ± 0.33^*^&^	62.82 ± 11.63^*^&^	79.92 ± 18.61^*^&^

Notice: *WT*, wild-type; *LF*, low fluoride; *HF*, high fluoride; *APP/PS1*, amyloid precursor protein/presenilin 1; *pr*, protein. The values presented are *P < 0.05 vs control (WT); ^#^*P* < 0.05 vs LF; ^^^*P* < 0.05 vs APP/PS1; ^&^*P* < 0.05 vs APP/PS1+LF, as determined by analysis of variance (ANOVA), followed by the least significant differences post hoc test

### The changes of these detected parameters in gender difference

No significant differences were detected in the fluoride contents of bones and brains, the levels of oxidative stress in brains, and the ability of learning and memory of the mice between female and male with APP/PS1 mutation and APP/PS1 mutation plus fluoride exposure (Table 4). Simultaneously, no changes were found in the parameters of APP metabolism and synaptic proteins between the gender difference from the mice with APP/PS1 mutation and APP/PS1 mutation plus fluoride exposure (Table 5).

**Table 4:** Biochemical parameters between genders of mice in different groups

Parameters	APP/PS1		APP/PS1+LF		APP/PS1+HF	
	Male	Female	Male	Female	Male	Female
Fluoride contents						
Bones (ppm)	1.01 ± 0.11	1.00 ± 0.12	1.35 ± 0.05	1.29 ± 0.03	2.51 ± 0.06	2.57 ± 0.02
Brains (ppm)	0.11 ± 0.01	0.11 ± 0.01	0.23 ± 0.02	0.26 ± 0.03	0.30 ± 0.01	0.29 ± 0.02
Morris water maze test						
Escape latency (s)	32.59 ± 3.34	34.06 ± 3.85	51.98 ± 3.76	54.65 ± 4.53	53.90 ± 2.34	51.98 ± 2.00
Number of time of crossing the platform (*N*)	2.00 ± 0.71	2.20 ± 0.84	0.20 ± 4.50	0.40 ± 0.55	0.60 ± 0.89	0.20 ± 0.45
Time remaining of the platform (s)	12.68 ± 1.27	12.89 ± 1.46	7.22 ± 0.96	7.32 ± 0.89	6.93 ± 1.05	7.45 ± 1.06
Number of senile plaques (*N*)	10.80 ± 1.79	11.80 ± 2.17	33.80 ± 3.70	38.00 ± 2.45	43.20 ± 3.39	45.00 ± 3.87
Oxidative stress						
MDA (nmol/mg pr)	4.71 ± 0.15	4.83 ± 0.10	6.60 ± 0.40	7.00 ± 0.48	7.34 ± 0.22	7.60 ± 0.49
SOD (U/mg pr)	121.29 ± 7.33	126.97 ± 5.67	80.31 ± 3.76	82.78 ± 4.59	59.18 ± 12.97	66.47 ± 9.45
GSH-Px (kU/l)	186.80 ± 5.78	193.18 ± 4.87	122.44 ± 8.17	129.66 ± 7.63	71.08 ± 9.53	87.86 ± 16.54

Notice: *APP/PS1*, amyloid precursor protein/presenilin 1; *pr*, protein. The values shown are means ± SD (*n* = 5), as determined by analysis of variance (ANOVA), followed by the least significant differences post hoc test

**Table 5:** Parameters in APP metabolism and synaptic proteins between genders of mice in different groups

	APP/PS1		APP/PS1+LF		APP/PS1+HF	
	Male	Female	Male	Female	Male	Female
BACE1	0.51 ± 0.20	0.49 ± 0.40	0.70 ± 0.30	0.62 ± 0.25	0.77 ± 0.20	0.80 ± 0.75
ADAM10	0.53 ± 0.20	0.47 ± 0.10	0.24 ± 0.10	0.36 ± 0.15	0.30 ± 0.20	0.23 ± 0.25
BACE2	0.43 ± 1.10	0.57 ± 0.75	0.44 ± 1.20	0.54 ± 0.90	0.37 ± 0.35	0.46 ± 0.50
SNAP-25	0.43 ± 0.10	0.57 ± 0.10	0.50 ± 0.95	0.41 ± 0.15	0.28 ± 0.30	0.37 ± 0.25
SYP	0.54 ± 0.15	0.46 ± 0.15	0.36 ± 0.10	0.46 ± 0.10	0.33 ± 0.05	0.29 ± 0.10

Notice: *APP/PS1*, amyloid precursor protein/presenilin 1; *LF*, lower fluoride; *HF*, high fluoride. The values shown are means ± SD (*n* = 5) as determined by analysis of variance (ANOVA), followed by the least significant differences post hoc test

## Discussion

The protocol employed here successfully led to chronic fluorosis in mice, as reflected in the elevated levels of fluoride in the brain and bone, and obvious dental fluorosis. The maximum contaminant level of F set by the World Health Organization (WHO) is 1.5 mg/l (1.5 ppm) [34]. However, it is estimated that more than 200 million people worldwide are dependent on drinking water with a fluoride level exceeding the criterion. The recommended fluoride uptake for humans is 0.05 mg/kg body weight per day with a tolerable upper intake of 10 mg/kg per day [35]. The fluoride dose for mice that is equivalent to humans can be more appropriately calculated by using a formula based on the body surface areas of mice and humans [36]. In the study here, we first prepared the stock solution (1 mg/l or 10 mg/l) as standard and the solutions were diluted by 10 times, respectively, when fed to animals with 0.3 ml by intragastric administration each day. Therefore, the doses of fluoride exposed to mice were equivalent to 1.5 ppm (close to the drinking water standard set by WHO) and 15 ppm, respectively, in drinking water for humans.

In previous studies by our group, we observed that the rats with chronic fluorosis exhibited a significantly declined ability of learning and memory [24, 25]. In the behavioral test, both the WT and APP/PS1 double-transgenic mice exposed to a high level of fluoride performed more poorly than the controls, indicating that impairment of spatial learning and memory. Prolonged exposure to the lower dose of fluoride significantly disrupted the learning and memory of mice carrying the APP/PS1 mutation only.

Senile plaques, spherical lesions containing extracellular aggregates of Aβ, result from the processing of APP by two proteases, β-secretase (e.g., BACE1) and γ-secretase. The two main products are Aβ_40_, which consists of the first 40 residues of APP, and Aβ_42_, containing the first 42 residues [37]. This production of Aβ can be avoided by alternate cleavage of APP first by the α-secretase, followed by the γ-secretase [5]. ADAM10 is generally considered to be the most active α-secretase in brain tissue [38] and activation of this enzyme in a murine model of AD suppresses production of Aβ [39].

Here, we found that APP/PS1 double-transgenic mice exhibited large numbers of extracellular amyloid plaques and accumulation of intracellular Aβ in their brains, in accordance with previous studies [40]. No such amyloid pathology was observed in our WT mice. Furthermore, semi-quantitative immunohistochemical analysis revealed that the number of senile plaques in the hippocampus of the APP/PS1 mice was significantly enhanced by either high- or lower-fluoride exposure. In addition, we further measured Aβ42 levels by ELISA and found the similar changes of Aβ42 levels in the cortex of mouse brains with APP/PS1 mutation exposed to lower or high fluoride.

BACE1 has been identified as a potential target for prevention of AD [41]. The activity of this secretase is correlated with the level of Aβ deposition in the brain, suggesting its involvement in the pathological production of Aβ [42]. Moreover, ADAM10s plays a critical role in attenuating Aβ production [43, 44]. Although the levels of BACE1, BACE2, and ADAM10 proteins were not shown significant changes in the brains of APP/PS1 and WT mice at the experiment stage, exposure to either of these groups to a high level of fluoride elevated the level of BACE1. In addition, exposure to either level of fluoride inhibited the expression of ADAM10 in the brains of APP/PS1 mice. The findings indicate that fluoride might promote BACE1 and suppress ADAM10 activity, thereby resulting in more Aβ production in the brains of the animals carrying the APP mutation.

Synaptic dysfunction is a major pathophysiological hallmark of AD, as well as other neurodegenerative diseases [45, 46]. Accumulating evidence indicates that the number of synapses in patients with AD or amnestic mild cognitive impairment, a predromal stage of AD, are reduced [47–49]. In addition, synaptic dysfunction is an early event in the pathogenesis of AD [50] and synaptic loss demonstrates the strongest correlation to dementia in these patients [51]. Our present results demonstrate the generalized loss of two synaptic proteins, SNAP-25 and SYP, in the brains of APP/PS1 mice. Moreover, in both these and WT mice fluoride at either dose reduced the levels of these synaptic proteins, which is consistent with past findings [52]. Inflammation and complement activation have emerged as a key event in the progression of synapse loss [53, 54]. Here, we investigated whether microgliosis and complement activation is responsible for the loss of synapse induced by fluoride exposure. Our current results showed that Iba-1, a microgliosis marker, was highly expressed in the APP/PS1 mice as compared to the WT control; in addition, with high- or lower-fluoride exposure enhanced the expression of Iba-1 in the brains of the mice with APP/PS1 mutation, suggesting that microglia-mediated inflammation induced by fluoride exposure might lead to the loss of synapse. While, no such changes of complement C3 among the different groups was observed in the present study.

Oxidative stress is often defined as an imbalance between free radical formation and their removal by cellular anti-oxidative systems, which leads to accumulation of toxic oxy-radicals, a process that plays a key role in both fluorosis and AD [55–58]. In recent years, it has been recognized that higher concentrations of fluoride result in neuronal dysfunction by elevating levels of free radicals while reducing the activities of antioxidant enzymes and contents of antioxidant compounds [24, 25]. Oxidative stress damages all classes of organic molecules in the cells, causing excessive lipid peroxidation and protein oxidation in the brains of patient with AD [59, 60]. Here, we found that both the APP/PS1 double-transgenic mutation and exposure to fluoride elevated the MDA content and reduced the activities of the antioxidant enzymes, SOD and GSH-Px in the brains of mice. Interestingly, exposure of fluoride significantly enhanced the level of oxidative stress in the brains of the mice with APP mutation.

Epidemiological studies showed an increase risk of AD with age-related loss of sex steroid hormones [61], which may be one of the causes of differences between men and women and their susceptibility to AD. In addition, the mice with APP/PS1 double-transgenic mutation exhibited a sex difference in the neuropathological changes [62]. However, in a study relating whether the progression of amyloidosis differentially affects males and females along aging in AβPP/PS1 transgenic mice, no significant differences of the Aβ burden in the cortex between the males and females aged from 3 to 12 months, although sex differences were more prominent in Aβ levels in the blood of older mice [63]. Furthermore, the accumulation of amyloid in the cerebellum differentially affected males and females of the APP/PS1 transgenic line, which was found to be tenfold higher in 15-month-old females [64]. In the study here, we did not find any significant differences among the biochemical parameters and the parameters of APP metabolism and synaptic proteins of the mice with APP/PS1 mutation and the exposure of fluoride between the female and male.

Appropriate fluoride exposure and usage is beneficial to tooth integrity and, as such, has an important, positive impact on health throughout life [65], while water fluoridation also brings about negative impact such as the chronic toxicity of fluoride [66]. Chronic fluorosis has been found to cause damage to the central nervous system and cognitive impairment [20, 21, 24–27, 67], but the relationship between fluoride and AD is still elusive. In present study here, we found that long-term exposure to fluoride (especially exposure to low fluoride) could enhanced the deficit in learning and memory and raised the numbers of senile plaque in mice carrying APP/PS1 double-transgenic mutation, which suggesting that long-term exposure to fluoride may be considered as risk factor of in development of AD.

## Conclusions

Our current observations indicated that in mice carrying APP/PS1 double-transgenic mutation, even lower levels of fluoride enhanced their deficit in learning and memory, raised the numbers of senile plaque, increased Aβ42 levels, modified the activities of enzymes that cleave APP, decreased the levels synaptic proteins, and elevated oxidative stress or inflammation in the brain. The result suggests that prolonged exposure to fluoride may accelerate the neuropathological lesions that occur in such APP mice.
